# Evaluation of the Incidence of Hematologic Malignant Neoplasms Among Breast Cancer Survivors in France

**DOI:** 10.1001/jamanetworkopen.2018.7147

**Published:** 2019-01-18

**Authors:** Marie Joelle Jabagi, Norbert Vey, Anthony Goncalves, Thien Le Tri, Mahmoud Zureik, Rosemary Dray-Spira

**Affiliations:** 1 University of Paris Sud, Paris-Saclay University, Paris, France; 2 Health Product Epidemiology Department, French National Agency for Medicines and Health Products Safety, Saint-Denis, France; 3 Aix-Marseille University, CNRS, Inserm, Institut Paoli-Calmettes, Hematology Department, CRCM, Marseille, France; 4 Aix-Marseille University, CNRS, Inserm, Institut Paoli-Calmettes, Medical Oncology Department, CRCM, Marseille, France; 5 Versailles Saint-Quentin-en-Yvelines University, Montigny-Le-Bretonneux, AP-HP Hôpital Sainte Perine Hospital, Paris, France

## Abstract

**Importance:**

Breast cancer survivors are at an increased risk of developing certain types of hematologic malignant neoplasm after diagnosis.

**Objective:**

To estimate the incidence of various types of hematologic malignant neoplasm in breast cancer survivors, both in absolute terms and in association with the general population.

**Design, Setting, and Participants:**

This nationwide cohort study conducted in France used data from the French National Health Data System, a database that contains all of French residents’ health-related expenses. All French women aged 20 to 85 years with an incident breast cancer diagnosis between July 1, 2006, and December 31, 2015, were included (n = 439 704) and followed up until hematologic malignant neoplasm occurrence, death, loss of follow-up, or December 31, 2016, whichever came first. Comparisons were made with all French women in the general population who were registered in the French general health insurance program each year from January 1, 2007, and December 31, 2016. Data analysis was performed from January 23, 2018, to May 25, 2018.

**Main Outcomes and Measures:**

Main outcomes were incident hematologic malignant neoplasm cases occurring at least 6 months after breast cancer diagnosis. The various types of hematologic malignant neoplasm considered were acute myeloid leukemia (AML), myelodysplastic syndrome (MDS), myeloproliferative neoplasms (MPNs), multiple myeloma (MM), Hodgkin lymphoma or non–Hodgkin lymphoma (HL/NHL), and acute lymphoblastic leukemia or lymphocytic lymphoma (ALL/LL). Incidence of these various types was estimated among breast cancer survivors and compared with the incidence in women in the general population.

**Results:**

The 439 704 women in the study had a median (interquartile range [IQR]) age of 59 (50-69) years and were followed up for a median (IQR) duration of 5 (2.8-7.5) years. Overall, 3046 cases of hematologic malignant neoplasm occurred: 509 cases (16.7%) of AML (crude incidence rate [CIR] per 100 000 person-years, 24.5; 95% CI, 22.4-26.8), 832 cases (27.3%) of MDS (CIR, 40.1; 95% CI, 37.4-42.9), and 267 cases (8.8%) of MPN (CIR, 12.8; 95% CI, 11.4-14.5). Lymphoid neoplasm cases included 420 cases (13.8%) of MM (CIR, 20.3; 95% CI, 18.4-22.3), 912 cases (29.9%) of HL/NHL (CIR, 44.4; 95% CI, 41.1-50.0), and 106 cases (3.5%) of ALL/LL (CIR, 5.1; 95% CI, 4.2-6.2). Compared with the general population, breast cancer survivors had statistically significantly higher incidence of AML (standardized incidence rate ratio [SIRR], 2.8; 95% CI, 2.5-3.2) and MDS (SIRR, 5.0; 95% CI, 4.4-5.7) and, to a lesser extent, MM (SIRR, 1.5; 95% CI, 1.3-1.7]) and ALL/LL (SIRR, 2.0; 95% CI, 1.3-3.0).

**Conclusions and Relevance:**

The finding that AML and MDS still occur among breast cancer survivors today, and that ALL/LL and MM may also be of concern, merits the continuous monitoring of hematologic malignant neoplasms and the thorough investigations into their underlying mechanisms.

## Key Points

**Question:**

Which hematologic malignant neoplasm types are more prone to develop after breast cancer diagnosis?

**Findings:**

In this cohort study of 439 704 French women, breast cancer survivors with an incident breast cancer diagnosis had statistically significantly higher standardized incidence rate ratios of acute myeloid leukemia and myelodysplastic syndrome compared with women in the general population. A slight increase in the incidence of multiple myeloma and acute lymphoblastic leukemia was noted.

**Meaning:**

This study sugests that acute myeloid leukemia, myelodysplastic syndrome, and acute lymphoblastic leukemia occur more frequently among breast cancer survivors than among women in the general population; continuous monitoring and further investigations into the underlying mechanisms of hematologic malignant neoplasms are necessary.

## Teaser

This cohort study study examines the incidence of secondary hematologic malignant neoplasms among French women after breast cancer diagnosis or treatment.

## Introduction

Secondary malignant neoplasms, including hematologic malignant neoplasms, that develop for months or years after the diagnosis of a primary tumor, are increasingly becoming a concern given that the population of breast cancer survivors is growing substantially. Such secondary cancers, reported since the early 1980s,^1,2,3,4^ may be a consequence of genetic predisposition, environmental factors, previous malignant neoplasm treatments, or an interaction among all those factors.^5,6^ Breast cancer is classified as the most prevailing malignant solid tumor associated with the risk of myeloid neoplasm development.^3,7,8^

Previous studies reporting a high risk of secondary myeloid neoplasm after breast cancer treatment were based on data from clinical trials, registries, or hospital series^4,9,10^ and addressed only acute myeloid leukemia and myelodysplastic syndrome. The risk of other hematologic malignant neoplasms after breast cancer treatment remains rarely studied.^2^ Little is known about the risk of lymphoid neoplasm secondary to a primary breast tumor. In addition, real-life data on secondary hematologic malignant neoplasm incidence are scarce, especially in the recent period marked by major advances in breast cancer treatments.

The aim of this study was to estimate the incidence of various hematologic malignant neoplasm types in breast cancer survivors during the past decade, including myeloid and lymphoid neoplasms, using real-life data, both in absolute terms and in association with French women in the general population.

## Methods

### Data Source

The French Data Protection Supervisory Authority approved this study. No informed consent was required because the data used in the study are anonymous and deidentified. This study followed the Strengthening the Reporting of Observational Studies in Epidemiology (STROBE) reporting guideline.

Data from the French National Health Data System (SNDS) were used. The SNDS database is unique in that it includes all of French residents’ health-related expenses. In France, each individual has a health insurance coverage plan, which varies according to the person’s occupational status. The general insurance plan covers both private and public sector employees as well as students, thus accounting for approximately 88% of the French population.

In the SNDS database, an anonymous unique subject identifier links information from different data sources: PMSI (the national hospital and discharge database), DCIR (the national health insurance claims database), and CepiDC (the national exhaustive database for the medical causes of death).

The PMSI database contains details of all private and public hospital admissions and discharges for either inpatient stays or ambulatory care. Data on diagnoses, treatments, and surgical procedures provided during hospital stays are also accessible.

The DCIR database includes individual information on sociodemographic characteristics, outpatient medical care, laboratory tests, and dispensed drugs. It also contains information about the presence of any severe or costly medical condition (per the *International Classification of Disease, Tenth Revision* [*ICD-10*] codes). Numerous international scientific publications and epidemiologic studies have used these databases.^11,12,13,14,15^

### Study Population

Breast cancer survivors were women aged 20 to 85 years who had an incident primary breast cancer diagnosis between July 1, 2006, and December 31, 2015, and who were registered in the general health insurance coverage program. Women were defined as having had breast cancer if they were eligible for 100% health insurance coverage for a severe or costly long-term disease with a diagnosis of invasive breast cancer (*ICD-10* code C50) or in situ breast cancer (*ICD-10* code D05) or if they had a hospital discharge diagnosis that included these codes. To restrict the study to incident cases of primary breast cancer, we excluded patients with a history of any type of cancer (the details of the *ICD-10* codes used can be found in eTable 1 in the Supplement). The breast cancer *diagnosis date* was defined as the earliest date between breast cancer onset as registered for severe and costly long-term disease coverage (if applicable) and the first hospital discharge diagnosis of breast cancer. Individuals who developed a hematologic malignant neoplasm in the first 6 months after breast cancer diagnosis were excluded. Breast cancer survivors were included in the study as of the breast cancer diagnosis date and were followed up until the occurrence of a hematologic malignant neoplasm, death, loss of follow-up (defined as 6 months after the last reimbursement), or December 31, 2016, whichever came first.

Women in the general population considered in the study were all French women aged 20 to 85 years and were registered in the general health insurance coverage program each year from 2007 to 2016.

### Outcome Identification

The main outcomes were incident hematologic malignant neoplasm cases that occurred at least 6 months after breast cancer diagnosis and were identified using *ICD-10* codes. Various types of myeloid and lymphoid neoplasms were considered. Myeloid neoplasm types comprised acute myeloid leukemia (AML), myelodysplastic syndrome (MDS), and myeloproliferative neoplasms (MPNs). Lymphoid neoplasm types comprised multiple myeloma (MM), Hodgkin lymphoma or non–Hodgkin lymphoma (HL/NHL), and acute lymphoblastic leukemia or lymphocytic lymphoma (ALL/LL). Detailed definitions of the outcomes, including their *ICD-10* codes, are presented in eTable 2 in the Supplement.

### Covariates

Sociodemographic characteristics included age and affiliation to complementary universal health insurance. Universal health insurance offers full free health care to people with low income. Lifestyle habits included alcohol use disorder, smoking-related conditions, and obesity. Medication history included hormone replacement therapy and medical contraception (eg, intrauterine device, birth control pills, and implant).

Breast cancer diagnosis modalities (mammography, biopsy, ultrasound, magnetic resonance imaging, and scans) were identified using information available within 45 days before or after breast cancer diagnosis. Information on breast cancer type (invasive or in situ) and treatment (surgical procedure, radiation therapy, chemotherapy, hormone therapy, targeted therapy, or growth factors) was also available. The codes and definitions of covariates can be found in eTable 3 in the Supplement.

### Statistical Analysis

Among breast cancer survivors, the crude incidence rates (CIRs) of each hematologic malignant neoplasm type after breast cancer diagnosis were estimated. Annual incidence rates by hematologic malignant neoplasm type were computed up to 10 years after breast cancer diagnosis (actuarial method).

For all women in the general population, crude overall and annual incidence rates were computed for each type of hematologic malignant neoplasm between January 1, 2007, and December 31, 2016. All incidence rates were age- and sex-specific, and 95% CIs were calculated using the exact method^16^ and assuming that the events followed a Poisson distribution. Time trends in incidence rates were examined using log linear Poisson regression to detect any change that could have occurred during this period.

Considering the age structure of the 2016 French population as a reference, we estimated, using direct standardization, the age-standardized incidence rates of each type of hematologic malignant neoplasm among breast cancer survivors and women in the general population. We computed standardized incidence rate ratios (SIRRs) with 95% CIs using Wald confidence limits. We also conducted a sensitivity analysis restricted to incident hematologic malignant neoplasm cases arising at least 1 year after breast cancer diagnosis.

Analyses were performed using SAS, version 9.4 (SAS Institute Inc). Data analysis was performed from January 23, 2018, to May 25, 2018.

## Results

### Breast Cancer Survivors

**Figure 1.**
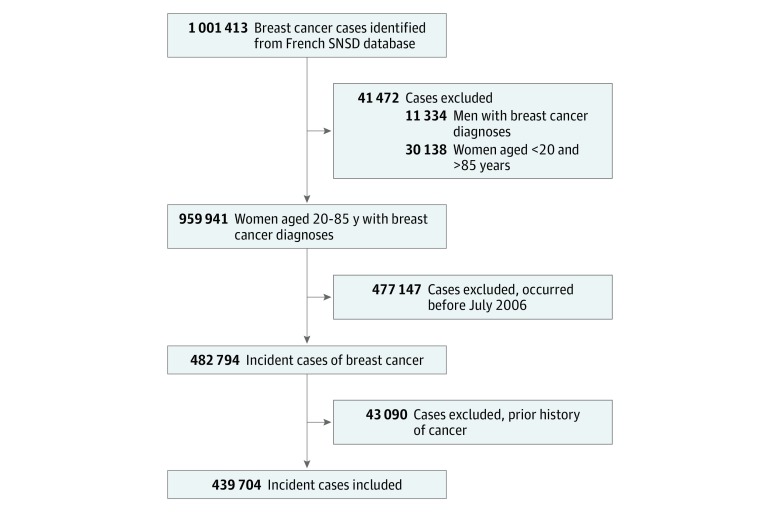
Study Population Flow Diagram SNDS is the French National Health Data System.

Of the total 1 001 413 cases of breast cancer identified in the SNDS database, 482 794 (48.2%) were incident cases that occurred between July 1, 2006, and December 31, 2015 among women aged 20 to 85 years. From the 482 794 incident cases, 43 090 women (8.9%) were excluded for having a personal history of other types of cancer. Thus, 439 704 women, with a median (interquartile range [IQR]) age of 59 (50-69) years were included in the cohort (Figure 1).

The median (IQR) follow-up time was 5 (2.8-7.5) years. The survival rates were 89% at 5 years and 79.3% at 10 years, with 53 388 patients (12.1%) dying during the follow-up period. As shown in Table 1, 19 376 patients (7.2%) had complementary universal health insurance. In total, 409 523 breast cancers (93.1%) were invasive and 30 181 (6.9%) were in situ. The most frequent methods of diagnosing breast cancer were mammography (89 427 [95.5%]), biopsy (87 182 [93.1%]), and ultrasonography (87 075 [93.0%]); other techniques, such as magnetic resonance imaging (29 506 [31.5%]) and positron emission tomography scanning (1332 [1.4%]), were used less frequently. Breast cancer was predominantly treated by surgical procedure (365 318 [83.1%]), radiation therapy (334 678 [76.1%]), and hormone therapy (291 007 [66.2%]) and less commonly by chemotherapy (186 142 [42.3%]). Anti–HER2 treatments were administered to 41 363 patients (9.4%) and growth factors were administered to 118 692 patients (27.0%) (Table 1).

**Table 1.** Characteristics of the Study Population

Variable	No. (%)
Age, median (IQR), y	59 (50-69)
Year of breast cancer diagnosis	
July-December 2006	21 841 (5.0)
2007-2009	138 482 (31.5)
2010-2012	139 592 (31.7)
2013-2015	139 789 (31.8)
Affiliation with complementary universal health insurance^a^	19 376 (7.2)
Comorbidities	
Measurable history of smoking	24 923 (5.7)
Alcohol use disorder	7113 (1.6)
Obesity	56 174 (12.8)
Medical contraception^b^	
IUD	24 260 (22.7)
Birth control pill (reimbursed by health insurance)	12 153 (11.4)
Birth control implant	1676 (1.6)
Hormone replacement therapy^c^	47 674 (12.7)
Breast cancer type	
Invasive	409 523 (93.1)
In situ	30 181 (6.9)
Breast cancer diagnosis^d^	
Mammography	89 427 (95.5)
Biopsy	87 182 (93.1)
Ultrasonography	87 075 (93.0)
MRI	29 506 (31.5)
PET scan	1332 (1.4)
Breast cancer treatment^e^	
Surgical procedure	365 318 (83.1)
Radiation therapy	334 678 (76.1)
Chemotherapy	186 142 (42.3)
Hormone therapy	291 007 (66.2)
Anti–HER2	41 363 (9.4)
Growth factors (antianemic/hematopoietic)	118 692 (27.0)

Abbreviations: IQR, interquartile range; IUD, intrauterine device; MRI, magnetic resonance imaging; PET, positron emission tomography.

^a^ Free access to health care for people with an annual income less than 50% of the poverty threshold and younger than 65 years (n = 169 031).

^b^ For patients younger than 50 years (n = 107 004).

^c^ For patients aged 45 years or older (n = 374 208).

^d^ For patients included in 2014-2015 (n = 93 615).

^e^ For any corresponding reimbursement registered within 1 year of cohort entry.

### Hematologic Malignant Neoplasms After Diagnosis

Overall, 3046 cases of hematologic malignant neoplasm occurred among breast cancer survivors. Cases of myeloid neoplasm consisted of the following types: 509 cases (16.7%) of AML (crude incidence rate [CIR] per 100 000 person-years, 24.5; 95% CI, 22.4-26.8), 832 cases (27.3%) of MDS (CIR, 40.1; 95% CI, 37.4-42.9), and 267 cases (8.8%) of MPN (CIR, 12.8; 95% CI, 11.4-14.5). Lymphoid neoplasm cases included 420 cases (13.8%) of MM (CIR, 20.3; 95% CI, 18.4-22.3), 912 cases (29.9%) of HL/NHL (CIR, 44.4; 95% CI, 41.1-50.0), and 106 cases (3.5%) of ALL/LL (CIR, 5.1; 95% CI, 4.2-6.2). Among women with hematologic malignant neoplasm, the median (IQR) age at breast cancer diagnosis ranged from 59.5 (50-69) years for ALL/LL to 72 (62-79) years for MDS. The median (IQR) time from breast cancer diagnosis to hematologic malignant neoplasm occurrence ranged from 2.4 (1.4-4.0) years for AML to 3.3 (1.8-5.5) years for MPN (Table 2).

**Table 2.** Crude Incidence Rates and Characteristics of Hematologic Malignant Neoplasm Types Among Breast Cancer Survivors

Type	Cases, No. (%) (n = 3406)	Crude Incidence Rate per 100 000 Person-Years (95% CI)	Median (IQR), y	
			Age at Breast Cancer Diagnosis	Time From Breast Cancer Diagnosis to Hematologic Malignant Neoplasms
Myeloid neoplasm				
Acute myeloid leukemia	509 (16.7)	24.5 (22.4-26.8)	63 (54-72)	2.4 (1.4-4.0)
Myelodysplastic syndrome	832 (27.3)	40.1 (37.4-42.9)	72 (62-79)	3.2 (1.5-5.2)
Myeloproliferative neoplasm	267 (8.8)	12.8 (11.4-14.5)	68 (58-75)	3.3 (1.8-5.5)
Lymphoid neoplasm				
Multiple myeloma	420 (13.8)	20.3 (18.4-22.3)	68 (59-75)	3.0 (1.3-4.9)
Hodgkin/non–Hodgkin lymphoma	912 (29.9)	44.4 (41.1-50.0)	68 (58-76)	3.2 (1.4-5.2)
Acute lymphoblastic leukemia/lymphocytic lymphoma	106 (3.5)	5.1 (4.2-6.2)	59.5 (50-69)	2.5 (1.3-4.0)

Abbreviation: IQR, interquartile range.

**Figure 2.**
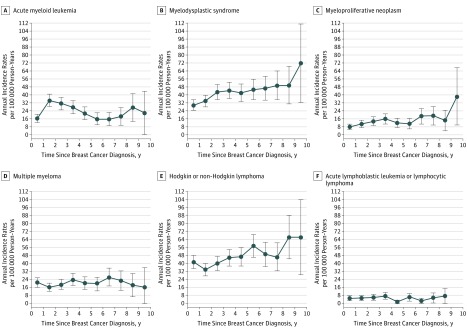
Annual Incidence Rates per 100 000 Person-Years for Each Hematologic Malignant Neoplasm Type Over Time Since Breast Cancer Diagnosis Each annual incidence rate is presented with a 95% CI (error bars).

Annual incidence rates of the different hematologic malignant neoplasm types, occurring after breast cancer diagnosis, showed different patterns over time (Figure 2). The AML incidence rate increased rapidly after breast cancer diagnosis, peaking during follow-up years 1 through 3 and then decreasing. A new peak was observed 7 to 9 years after breast cancer diagnosis. The incidence rates for MDS, MPN, and HL/NHL increased sustainably over time since breast cancer diagnosis, whereas MM and ALL/LL incidence rates remained stable during follow-up.

### Comparison With the General Population

Among women in the general population, 148 619 incident cases of hematologic malignant neoplasms were identified between 2007 and 2016. These cases consisted of the following types: 15 727 AML (10.6%; CIR per 100 000 person-year, 7.2; 95% CI, 7.1-7.3), 16 113 MDS (10.8%; CIR, 7.4; 95% CI, 7.3-7.5), 22 471 MPN (15.1%; CIR, 10.3; 95% CI, 10.2-10.4), 22 744 MM (15.3%; CIR, 10.4; 95% CI, 10.3-10.6), 66 346 HL/NHL (44.6%; CIR, 30.5; 95% CI, 30.2-30.7), and 5218 ALL/LL (3.5%; CIR, 2.4; 95% CI, 2.3-2.5). No statistically significant time trend in the incidence of any hematologic malignant neoplasm type was observed between 2007 and 2016. None of the small increasing or small decreasing annual percentage change of MN incidence was statistically significant (eFigure in the Supplement).

**Figure 3.**
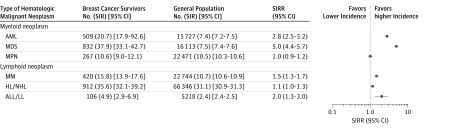
Forest Plot of Standardized Incidence Rate Ratios (SIRRs) of Hematologic Malignant Neoplasm Types Among Breast Cancer Survivors Compared With French Women in the General Population All standardized incidence rates (SIRs) are presented with 95% CIs. No. indicates the number of events in each population. ALL/LL indicates acute lymphoblastic leukemia or lymphocytic lymphoma; AML, acute myeloid leukemia; HL/NHL, Hodgkin or non–Hodgkin lymphoma; MDS, myelodysplastic syndrome; MM, multiple myeloma; MPN, myeloproliferative neoplasm.

After age standardization, the incidence of hematologic malignant neoplasm was statistically significantly higher among breast cancer survivors than among women in the general population for AML (SIRR, 2.8; 95% CI, 2.5-3.2), MDS (SIRR, 5.0; 95% CI, 4.4-5.7), MM (SIRR, 1.5; 95% CI, 1.3-1.7) and ALL/LL (SIRR, 2.0; 95% CI, 1.3-3.0). In contrast, the SIRRs of MPN (1.0; 95% CI, 0.9-1.2) and HL/NHL (1.1; 95% CI, 1.0-1.3) did not statistically significantly differ between breast cancer survivors and the general population (Figure 3). Sensitivity analysis restricted to hematologic malignant neoplasm cases that occurred more than 1 year after breast cancer diagnosis yielded similar results with a slight increase in the AML incidence (SIRR, 3.0; 95% CI, 2.6-3.5) and MDS incidence compared with the general population (SIRR, 3.2; 95% CI, 2.8-3.8) (eTable 4 in the Supplement).

## Discussion

This population-based study of 439 704 patients with an incident primary breast cancer from 2006 to 2016 with a median follow-up period of 5 years enabled us to estimate the incidence of various secondary hematologic malignant neoplasms in breast cancer survivors in real life. The incidence of AML, MDS, and, to a lesser extent, MM and ALL/LL appeared to be higher among breast cancer survivors than among French women in the general population.

The number of incident cases of breast cancer identified (439 706 over the study period, or a mean of approximately 46 000 per year) and their characteristics and prognosis (median age of 59 years at diagnosis, and 5-year overall survival higher than 85%) were consistent with figures reported in France and in other countries.^17,18^ The real-life data we found showed therapeutic and diagnostic practices that were similar to those reported in other studies.^19,20,21,22^ Hematologic malignant neoplasm incidence rates in the general population, which were estimated in this study on the basis of SNDS data, were comparable to the estimates based on data from national registries.^23^ In accordance with data reported by other countries, we did not find notable changes in the general population’s incidence of hematologic malignant neoplasm during this relatively short and recent period.^24,25,26^

We found a substantially elevated incidence of AML and MDS among breast cancer survivors. French women in the study having had an incident breast cancer in the past decade were nearly 3 times more likely to develop AML and 5 times more likely to develop MDS than women in the general population. Several studies linked AML and MDS to chemotherapeutic agents,^10,27,28^ radiation treatment,^29,30,31^ and supportive treatment with granulocyte colony-stimulating factor.^4^ These results are consistent with other available data showing a 2½-fold to 3½-fold increased risk of AML.^9,27,30^ Our estimates of a 5-fold increase in MDS risk were slightly higher compared with data reporting a 3.7-fold increased risk^9^; MDS risk might likely be underreported in other data sets.^32^

We cannot disregard that hematologic malignant neoplasm risk peaks within specific time frames. The annual incidence of AML among the breast cancer survivors in this study increased in the first few years after breast cancer diagnosis, with an early peak around the third year and a subsequent peak around the eighth year. This finding is consistent with previous reports suggesting the presence of 2 types of therapy-associated AML: 1 type associated with specific translocation such as t(15;17), t(8;21), or inv(16) occurring early (1-3 years) after exposure to topoisomerase inhibitors, and 1 type associated with myelodysplastic-related changes occurring 5 to 7 years after exposure to DNA-damaging agents.^4,33^ In our study, the median (IQR) time between breast cancer diagnosis and AML onset of 2.4 (1.4-4.0) years was shorter than the median (IQR) gap reported in other studies: 4 (0.3-44.1) years^34^ and 5.6 (0.5-38.4) years.^35^ Latency depends on the patient’s age at diagnosis, therapy type, and dose regimen,^34^ but this difference could be a consequence of the limited length of follow-up in the present study.

Most secondary leukemias are myeloid, but secondary ALLs are believed to constitute 10% to 12% of all secondary leukemias,^36^ with breast cancer being the most common primary malignant neoplasm at its origin. In this study, a 2-fold increase in incidence of ALL/LL was found among breast cancer survivors. The results pointed to an increase in the incidence of this poor prognosis outcome,^37,38^ despite ALL being rare. Sparse data are available in the literature, and secondary ALL is poorly characterized. Some studies have demonstrated that radiation^39^ and chemotherapy^40^ are associated with the pathogenesis, whereas other studies have suggested that prior therapy plays a less important role in secondary ALL than genetic predisposition.^41^

A 50% excess in MM incidence was observed among breast cancer survivors in this study. Such a slight increase has not been previously reported and needs to be investigated more thoroughly. In particular, the role of inherited susceptibility should be sorted out, given the increased risk of MM in carriers of the *BRCA1* (OMIM 600185) and *BRCA2* (OMIM 113705) gene mutations.^42^

### Strengths and Limitations

A strength of this study is the availability of a nationwide population-based data, including a complete panel of information on patients, their therapies and their hospital stays, along with a relatively long-term follow-up in the recent therapeutic era. The sample size allowed us to assess the incidence of various hematologic malignant neoplasm types after breast cancer diagnosis, including the rarest. To our knowledge, compared with other investigations, this study included by far the greatest number of hematologic malignant neoplasm events occurring after breast cancer diagnosis. The general population, with its specific incidence, served as a comparison group. Hematologic malignant neoplasm incidence rates in this comparison group were estimated for the same study period, using the same data source and the same outcome definition for all French women in the general population. We were able to measure the cumulative incidence of rare events to provide a post–breast cancer perspective based on real-life data.

The absence of an external clinical validation of the hematologic malignant neoplasm and breast cancer diagnosis remains a potential limitation of this study. Yet, our results were consistent with findings from other studies, and the hematologic malignant neoplasm incidence rates in the general population were comparable to those reported in national registries. In addition, many studies showed that the administrative data sets based on the *ICD-10* codes are accurate and can be used. A systematic review reported that most of these studies had a sensitivity of more than 80% and a specificity of more than 98% in breast cancer identification.^43^ Likewise, the hematologic malignant neoplasm incidence rates in the general population were comparable to those reported in registries.

In addition, the study lacked cytogenetic information that could have confirmed a therapy-associated malignant neoplasm or a genetic predisposition. However, the main objective of this study was not to establish causality but rather to estimate the increased incidence of hematologic malignant neoplasm in breast cancer survivors. Also, the study was restricted to women covered by the general health insurance scheme in France. However, because 88% of the French population is covered, this limitation is unlikely to generate selection bias or call into question the generalizability of this study’s results.

## Conclusions

Findings from this study suggest that, in the recent era, AML and MDS occur more frequently among breast cancer survivors than among women in the general population. Other hematologic malignant neoplasm types such as ALL/LL and MM may also be a concern for breast cancer survivors. These findings serve to better inform practicing oncologists, and breast cancer survivors should be advised of the increased risk of developing certain hematologic malignant neoplasms after their first cancer diagnosis. The recent discovery of the gene signatures that guide treatment decisions in early-stage breast cancer might reduce the number of patients exposed to cytotoxic chemotherapy and its complications, including hematologic malignant neoplasm.^44,45^ Therefore, continuing to monitor hematologic malignant neoplasm trends is necessary, especially given that approaches to cancer treatment are rapidly evolving. Further research is also required to assess the modality of treatment for and the genetic predisposition to these secondary malignant neoplasms.

## Notes

Supplement. **eTable 1.** ICD-10 Codes Used to Determine Patients With History of Cancer **eTable 2.** Detailed Definition of the Outcome Subtypes With Their ICD-10 Codes **eTable 3.** Details of Covariates **eTable 4.** Standardized Incidence Ratio and Rate Ratio of Hematological Malignancies Occurring More Than 1 Year After Breast Cancer Diagnosis **eFigure.** Secular Trend of Hematological Malignancy Incidence Rates in French Women of the General Population
