# Supplementary material for: Evaluation of the Incidence of Hematologic Malignant Neoplasms Among Breast Cancer Survivors in France
Source: JAMA Netw Open. 2019 Jan 18;2(1):e187147. doi: 10.1001/jamanetworkopen.2018.7147 (PMC6484549; doi:10.1001/jamanetworkopen.2018.7147)
Supplement: Supplement. — eTable 1. ICD-10 Codes Used to Determine Patients With History of Cancer eTable 2. Detailed Definition of the Outcome Subtypes With Their ICD-10 Codes eTable 3. Details of Covariates eTable 4. Standardized Incidence Ratio and Rate Ratio of Hematological Malignancies Occurring More Than 1 Year After Breast Cancer Diagnosis eFigure. Secular Trend of Hematological Malignancy Incidence Rates in French Women of the General Population [file jamanetwopen-2-e187147-s001.pdf]

Supplementary Online Content

Jabagi MJ, Vey N, Goncalves A, Tri TL, Zureik M, Dray-Spira R. Evaluation of the incidence of hematologic malignant neoplasms among breast cancer survivors in France. *JAMA Netw Open.* 2019;2(1): e187147. doi:10.1001/jamanetworkopen.2018.7147

eTable 1. ICD-10 Codes Used to Determine Patients With History of Cancer

eTable 2. Detailed Definition of the Outcome Subtypes With Their ICD-10 Codes

eTable 3. Details of Covariates

eTable 4. Standardized Incidence Ratio and Rate Ratio of Hematological Malignancies Occurring More Than 1 Year After Breast Cancer Diagnosis

eFigure. Secular Trend of Hematological Malignancy Incidence Rates in French Women of the General Population

This supplementary material has been provided by the authors to give readers additional information about their work.

© 2019 Jabagi MJ et al. *JAMA Network Open*.

eTable 1. ICD-10 Codes Used to Determine Patients With History of Cancer

**Exclusion Criteria**	**ICD-10 codes : Indication**
**History of cancer Indicators**	Anemia in neoplastic disease **(D63.0)**; Tumor lysis syndrome **(E88.3)**; Cranial nerve disorders in neoplastic diseases **(G53.3)**; Nerve root and plexus compressions in neoplastic diseases **(G55.0)**; Polyneuropathy in neoplastic diseases **(G63.1)**; Myasthenic syndromes in neoplastic diseases **(G73.2)**; Other disorders of brain in neoplastic diseases **(G94.1)**; Acute pulmonary manifestations due to radiation **(J70.0)**; Chronic and other pulmonary manifestations due to radiation **(J70.1)**; Gastroenteritis and colitis due to radiation **(K52.0)**; Radiation proctitis **(K62.7)**; Acute radiodermatitis **(L58.0)**; Chronic radiodermatitis **(L58.1)**; Other specified disorders of the skin and subcutaneous tissue related to radiation **(L59.8)**; Disorder of the skin and subcutaneous tissue related to radiation **(L59.9)**; Dermato(poly)myositis in neoplastic disease **(M36.0)**; Arthropathy in neoplastic disease **(M36.1)**; Osteitis deformans in neoplastic diseases **(M90.6)**; Post radiation kyphosis **(M96.2)**; Post radiation scoliosis **(M96.5)**; Irradiation cystitis **(N30.4)**; Maternal care for (suspected) damage to fetus by radiation **(O35.6)**; Poisoning by, adverse effect of and under dosing of antineoplastic and immunosuppressive drugs **(T45.1)**; Radiation sickness **(T66)**; Encounter for follow-up examination after completed treatment for malignant neoplasm **(Z08).**
**Cancer diagnosis**	Malignant neoplasms **(C00-C97)**; Carcinoma in Situ **(D00-D09)**; Benign neoplasm **(D10-D36)**; Neoplasm of uncertain behavior of other and unspecified sites **(D37-D48).**
**History of cancer treatment**	Radiotherapy session **(Z51.0)**; Chemotherapy session for neoplasm **(Z51.1)**.

Abbreviation: ICD-10, International Classification of diseases, tenth revision codes.

© 2019 Jabagi MJ et al. *JAMA Network Open*.

eTable 2. Detailed Definition of the Outcome Subtypes With Their ICD-10 Codes

**Hematological Malignancies**		**Subtypes (ICD -10 codes)**
**Myeloid Neoplasm**	Acute myeloid leukemia (AML)	Acute myeloid leukemia **(C92.0/C92.8/C92.4/C92.5/C92.6)**<br>Acute myeloid promyelocytic leukemia **(C92.4)**
	Myelodysplastic Syndrome (MDS)	Myelodysplastic Syndrome **(D46)**
	Myeloproliferatif neoplasm (MPN)	Chronic myeloid leukemia **(C92.1)**<br>Chronic myeloproliferative disease **(D47.1)**
**Lymphoid Neoplasm**	Multiple Myeloma (MM)	Multiple myeloma and malignant plasma cell neoplasms **(C90)**
	Hodgkin and Non Hodgkin lymphoma (HL/NHL)	Hodgkin lymphoma **(C81)**<br>Chronic lymphocytic leukemia of B-cell type **(C91.1)**<br>Small cell B-cell lymphoma **(C83.0)**<br>Follicular lymphoma **(C82)**<br>Diffuse large B-cell lymphoma **(C83.3)**<br>Mantle cell lymphoma **(C83.1)**<br>MALT-lymphoma **(C88.4)**<br>Other specified types of T/NK-cell lymphoma **(C86)**<br>Other non-follicular lymphoma **(C83.8)**<br>Waldenström macroglobulinemia **(C88.0)**<br>Neoplasm of uncertain behavior of lymphoid and related tissue **(D47.9)**
	Acute Lymphoblastic Leukemia /Lymphoblastic Lymphoma (ALL/LL)	Acute lymphoblastic leukemia **(C91.0)**<br>Adult T-cell lymphoma/leukemia **(C91.5)**<br>Lymphoblastic (diffuse) lymphoma **(C83.5)**

Abbreviation: ICD-10, International Classification of diseases, tenth revision codes.

© 2019 Jabagi MJ et al. *JAMA Network Open*.

eTable 3. Details of Covariates

Covariates	Code Type	List of codes	Delay used for algorithm construction
**Measurable history of smoking**	ATC codes	N06AX12 N07BA	1 year before inclusion and at inclusion
	ICD-10 codes	F17 Z71.6 Z72.0 I731 J41 J42 J43 J44 T65.2	
**Obesity**	ATC codes	A08AB01, A08AX01, A10B	2 years before inclusion and at inclusion
	ICD-10 codes	Z68.3 Z68.4 E65 E66	
	Common classification of medical procedure codes	HFCA001 HFCC003 HFFA001 HFFA011 HFFC004 HFFC018 HFMA009 HFMC008 HFMA011 HFKA001 HFMA010 HFMC006 HFMC007 HGCA009 HGCC027 HFLE002 HFLC900 HFGC900 HFKC001 HFKA002	
**Alcohol use disorder**	ATC codes	N07BB01, N07BB02, N07BB03, N07BB04, N07BB05	1 year before inclusion and at inclusion
	ICD-10 codes	F10 K70 T51 E24.4 G31.2 G62.1 G72.1 I42.6 K29.2 K86.0 T51.0 T51.9 Z50.2 Z71.4 Z72.1	
**Oral contraceptive pill / implant, transdermal patch**	Common classification of medical procedure codes	QZGA001, QZGA002, QZGA003, QZGA004, QZGA006, QZGA007, QZGA008, QZGA010	Any reimbursement registered 6 months before cohort entry
	ATC codes	G02BB01, G03AA, G03AB, G03AC	
**Intrauterine Device**	Common classification of medical procedure codes	JKLD001, JKGA001, JKGC001, JKGD001, JKGD004, JKGE001, JKKD001	Until 5 years before inclusion
	ATC codes	G02BA01, G02BA02, G02BA03	
	List of reimbursed products and services codes	1101938, 1103848, 1106752, 1111760, 1120717, 1121125, 1122283, 1125749, 1128370, 1132519, 1132531, 1134760, 1135890, 1146770, 1152960, 1158536, 1167363, 1171407, 1173062, 1187615	

© 2019 Jabagi MJ et al. *JAMA Network Open*.

**Hormone replacement therapy**	ATC codes	G03XC G03CA G03CC G03CX G03CA G03CC G03CX G03FA G03FB G03FA G03FB G03HB G03CA G03CC G03CX G03FA G03FB G03HB	Any reimbursement registered 6 months before cohort entry
**Breast Cancer diagnosis procedures**	Common classification of medical procedure codes	QELH001 QEQK005 QEQH001 QEQK004 QEQK003 QELJ001 QEQM001 QEQH002 QEQK006 QEQJ001 QEQN001 QEHP002 QEHA001 QEHA002 QEHB00 QEHB002 QEHH001 QEHH002 QEHH003 QEHH015 QEHJ001 QEHJ002 QEHJ003 QEHJ004 QEHJ005 QEHJ006	45 days before and 45 days after inclusion
	ICD-10 codes	Z12 Z123 Z139 T812 Y606 Y848 T810 R92 Z016 Z123 Z031 Y848 I978	
**Breast Cancer treatment**			
**Surgery**	Common classification of medical procedure codes	QEFA019 QEFA017 QEFA008 QEFA003 QEFA007 QEFA005 QEFA010 QEFA020 QEFA015 QEFA013 QEFA012 QEFA016 QEFA001 QEFA004	Any reimbursement happening in the year after inclusion
**Radiotherapy**	Common classification of medical procedure codes	YYYY493 YYYY511 YYYY520 YYYY500 YYYY497 YYYY599 YYYY492 YYYY256 YYYY244 YYYY470 YYYY522 YYYY533 YYYY577 YYYY588 YYYY566 YYYY555 YYYY544 YYYY175 YYYY197 YYYY211 YYYY223 YYYY304 YYYY305 YYYY316 YYYY315 YYYY320 YYYY323 YYYY324 YYYY314 YYYY313 YYYY048 YYYY021 YYYY049 YYYY050 YYYY099 YYYY299 YYYY267 YYYY301 YYYY302 YYYY303 YYYY306 YYYY307 YYYY310 YYYY312 YYYY152 YYYY141 YYYY045 YYYY023 YYYY046 YYYY047 YYYY101 YYYY122 YYYY136 YYYY056 YYYY055 YYYY051 YYYY052 YYYY053 YYYY054 YYYY325 YYYY326 YYYY391 YYYY390 YYYY392 YYYY393 YYYY398 YYYY387 YYYY383 YYYY379 YYYY380 YYYY381 YYYY458 YYYY459 YYYY460 YYYY377 YYYY371 YYYY343 YYYY338 YYYY345 YYYY346 YYYY382 YYYY450 YYYY451 YYYY471	Any reimbursement happening in the year after inclusion

© 2019 Jabagi MJ et al. *JAMA Network Open*.

		YYYY479 YYYY480 YYYY481 YYYY469 YYYY468 YYYY457 YYYY347 YYYY337 YYYY336 YYYY327 YYYY331 YYYY334 YYYY335 YYYY348 YYYY349 YYYY367 YYYY368 YYYY369 YYYY370 YYYY365 YYYY360 YYYY356 YYYY357 YYYY358 YYYY359 YYYY491 ZZNL040 ZZNL042 ZZNL039 ZZNL037 ZZNL034 ZZNL036 ZZNL043 ZZNL045 ZZNL049 ZZNL050 ZZNL048 ZZNL047 ZZNL046 ZZNL033 ZZNL031 ZZNL020 ZZNL021 ZZNL016 ZANL001 ZZNL052 ZZNL023 ZZNL024 ZZNL028 ZZNL030 ZZNL027 ZZNL026 ZZNL025 ZZNL051 ZZNL904 ZZNL900 ZZNL903 ZZNL061 ZZNL066 ZZNL064 ZZNL065 ZZNL063 ZZNL062 ZZNL902 ZZNL053 ZZNL054 ZZNL060 ZZNL059 ZZNL055 ZZNL058 ZZNL906 ZZNL905	
	ICD-10 codes	Z510 Z541	
			-
**Anti HER2+**	ATC codes	L01XC13 L01XC14 L01XC03 L01XE07	Any reimbursement happening in the year after inclusion
			-
**Hormonotherapy**	ATC codes	L02BA01 L02BA02 L02BA03 L02BG03 L02BG06 L02BG04 L02AE03 L02AE02	Any reimbursement happening in the year after inclusion
			-
**Chemotherapy**	Common classification of medical procedure codes	HPLB007 HPLB002 GGLB001 HPLB003 ZZLF004 AFLB003 GGLB008 AFLB013 EDLF019 EBLF003 ECLF006 EELF004 EDLF020 EDLF015 EDLF014 EDLF018 EDLF021 EELF005 EBLF002 ECLF005 EDLF017 EDLF016 ABLB006 ZZLF900	Any reimbursement happening in the year after inclusion
	ICD-10 codes	Z511 Z542 Z292	

Abbreviation: ICD-10, International Classification of diseases, tenth revision codes; ATC, Anatomical Therapeutic Chemical Classification System Codes.

© 2019 Jabagi MJ et al. *JAMA Network Open*.

eTable 4. Standardized Incidence Ratio and Rate Ratio of Hematological Malignancies Occurring More than 1 Year After Breast Cancer Diagnosis

Type of HM	Number of cases	SIR (95%CI) [a]	SIRR (95%CI) [b]	Pval
**Myeloid neoplasms**				
Acute Myeloid Leukemia	485	22.3 (19.1-25.5)	3.0 (2.6-3.5)	<.001
Myelodysplastic Syndrome	779	40.1 (34.8-45.4)	5.3 (4.6-6.1)	<.001
Myeloproliferative Neoplasm	252	11.3 (9.5-13.0)	1.1 (0.9-1.3)	0.35
**Lymphoid Neoplasm**				
Multiple Myeloma	369	15.5 (13.5-17.4)	1.4 (1.3-1.6)	<.001
Hodgkin/Non hodgkin Lymphoma	812	35.4 (31.6-39.1)	1.1 (1.0-1.3)	0.02
Acute Lymphoblastic leukemia/Lymphoma	96	5.1 (2.9-7.4)	2.1 (1.4-3.3)	0.001

Abbreviations: HM = Hematological malignancies; [a] SIR, Standardized Incidence Rate; [b] SIRR = Standardized Incidence Rate Ratio;

© 2019 Jabagi MJ et al. *JAMA Network Open*.

eFigure. Secular Trend of Hematological Malignancy Incidence Rates in French Women of the General Population

Marrow Neoplams	CIR (95%CI)	APC (%) 95%CI	APC (Pvalue)
AML	7.2 (7.1-7.3)	1.41 [-4.6;7.7]	0.6
MDS	7.4 (7.3-7.5)	0.69 [-5.1;6.8]	0.8
MM	10.4 (10.3-10.6)	-0.7 [-8.0;7.2]	0.9
HL/NHL	30.5 (30.2-30.7)	2.16 [-3.9;8.6]	0.5
ALL/LL	2.4 (2.3-2.5)	-0.36 [-4.2;3.6]	0.8
MPN	10.3 (10.2-10.4)	1.37 [-5.4;8.7]	0.7

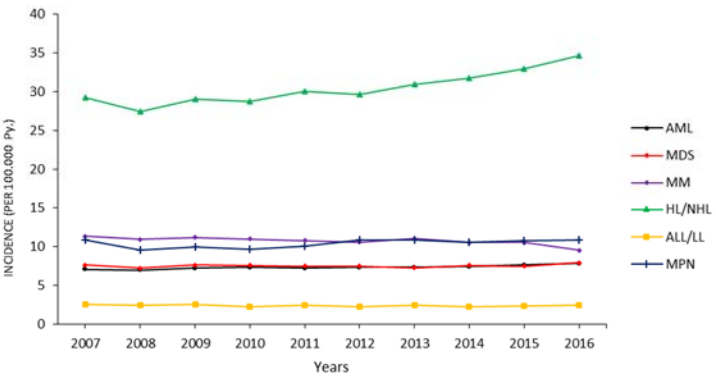

Abbreviations: AML, Acute Myeloid Leukemia; MDS, Myelodysplastic Syndrome; MPN, Myeloproliferative Neoplasms; MM, Multiple Myeloma; HL/NHL, Hodgkin and Non Hodgkin Lymphoma; ALL/LL, Acute Lymphoblastic Leukemia / Lymphoma; APC, annual percentage change; CIR, crude incidence rate.

© 2019 Jabagi MJ et al. *JAMA Network Open*.
